# A Preclinical Blinded Randomized-Controlled Trial Evaluating the Clinical Relevance of Polyp Size Measurement Using a Virtual Scale Endoscope

**DOI:** 10.1093/jcag/gwad057

**Published:** 2023-12-23

**Authors:** Daniel Kaufman, Roupen Djinbachian, Mahsa Taghiakbari, Ioana Popescu Crainic, Claire Haumesser, Maria Abou Khalil, Sacha Sidani, Jeremy Liu Chen Kiow, Benoit Panzini, Daniel von Renteln

**Affiliations:** Montreal University Hospital Research Center (CRCHUM), Departement of Gastroenterology, Montreal, QC, Canada; University of Montreal Medical School, Montreal, QC, Canada; Montreal University Hospital Research Center (CRCHUM), Departement of Gastroenterology, Montreal, QC, Canada; Department of Gastroenterology, Montreal University Hospital Center (CHUM), Montreal, QC, Canada; Montreal University Hospital Research Center (CRCHUM), Departement of Gastroenterology, Montreal, QC, Canada; Department of Gastroenterology, Montreal University Hospital Center (CHUM), Montreal, QC, Canada; Montreal University Hospital Research Center (CRCHUM), Departement of Gastroenterology, Montreal, QC, Canada; University of Montreal Medical School, Montreal, QC, Canada; Department of Internal Medicine, Montreal University Hospital Center (CHUM), Montreal, QC, Canada; Montreal University Hospital Research Center (CRCHUM), Departement of Gastroenterology, Montreal, QC, Canada; University of Montreal Medical School, Montreal, QC, Canada; Montreal University Hospital Research Center (CRCHUM), Departement of Gastroenterology, Montreal, QC, Canada; Department of Gastroenterology, Montreal University Hospital Center (CHUM), Montreal, QC, Canada; Montreal University Hospital Research Center (CRCHUM), Departement of Gastroenterology, Montreal, QC, Canada; Department of Gastroenterology, Montreal University Hospital Center (CHUM), Montreal, QC, Canada; Montreal University Hospital Research Center (CRCHUM), Departement of Gastroenterology, Montreal, QC, Canada; Department of Gastroenterology, Montreal University Hospital Center (CHUM), Montreal, QC, Canada; Montreal University Hospital Research Center (CRCHUM), Departement of Gastroenterology, Montreal, QC, Canada; Department of Gastroenterology, Montreal University Hospital Center (CHUM), Montreal, QC, Canada; Montreal University Hospital Research Center (CRCHUM), Departement of Gastroenterology, Montreal, QC, Canada; Department of Gastroenterology, Montreal University Hospital Center (CHUM), Montreal, QC, Canada

**Keywords:** colonoscopy, colorectal cancer, polyp size measurement, virtual scale

## Abstract

**Background:**

The virtual scale endoscope (VSE) helps endoscopists measure colorectal polyp size more accurately compared to visual assessment (VA). However, previous studies were not adequately powered to evaluate the sizing of polyps at clinically relevant size thresholds and relative accuracy for size subgroups.

**Methods:**

We created 64 artificial polyps of varied sizes and Paris class morphology, randomly assigned 1:1 to be measured (383 total measurement datapoints with VSE and VA by 6 endoscopists blinded to true size) in a colon model. We added data from two previous trials (480 measurement datapoints). We evaluated for correct classification of polyps into size groups at 3 mm, 5 mm, 10 mm, and 20 mm size thresholds and the relative size measurement accuracy for diminutive polyps (≤5 mm), small polyps (5–9 mm), large polyps at 10–19 mm, and polyps (≥20).

**Results:**

VSE had significantly less size group misclassifications at the 5 mm, and 10 mm thresholds (28 percent vs. 45 percent, *P* = 0.0159 and 26 percent vs. 44 percent, *P* = 0.0135, respectively). For the 3 mm and 20 mm thresholds, VSE had lower misclassifications; however, this was not statistically significant (36 percent vs. 46 percent, *P* = 0.3853 and 38 percent vs. 41 percent, *P* = 0.2705, respectively). The relative size measurement accuracy was significantly higher for VSE compared to VA for all size subgroups (diminutive (*P* < 0.01), small polyps (*P* < 0.01), 10–19 mm (*P* < 0.01), and ≥20 mm (*P* < 0.01)).

**Conclusion:**

VSE outperforms VA in categorizing polyps into size groups at the clinically relevant size thresholds of 5 mm and 10 mm. Using VSE resulted in significantly higher relative measurement accuracy for all size subgroups.

## Introduction

Currently, there is no gold standard for measuring polyp size during colonoscopy. Many methods, including biopsy forceps and snare instruments, can be used but data demonstrating measurement accuracy is sparse.^1^ Instead, endoscopists often rely on visual assessment (VA), which is prone to interobserver variability and inaccuracies.^2,3^ Recent advancements in colonoscope technology have led to the development of a virtual-scale endoscope (SCALE-EYE, Fujifilm, Tokyo, Japan). The virtual scale can measure polyp size during colonoscopy procedures. While previous studies, both pre-clinical and clinical, have demonstrated that the virtual scale endoscope (VSE) overall outperforms VA at measuring polyps accurately, limitations still exist. It is particularly difficult to conduct clinical studies adequately powered for all size subgroups. Thus, we conducted pre-clinical experiments to evaluate size measurement performance for all subgroups of sizes and performance of classification at size thresholds.

## Methods

### Experimental setup

**Figure 1.**
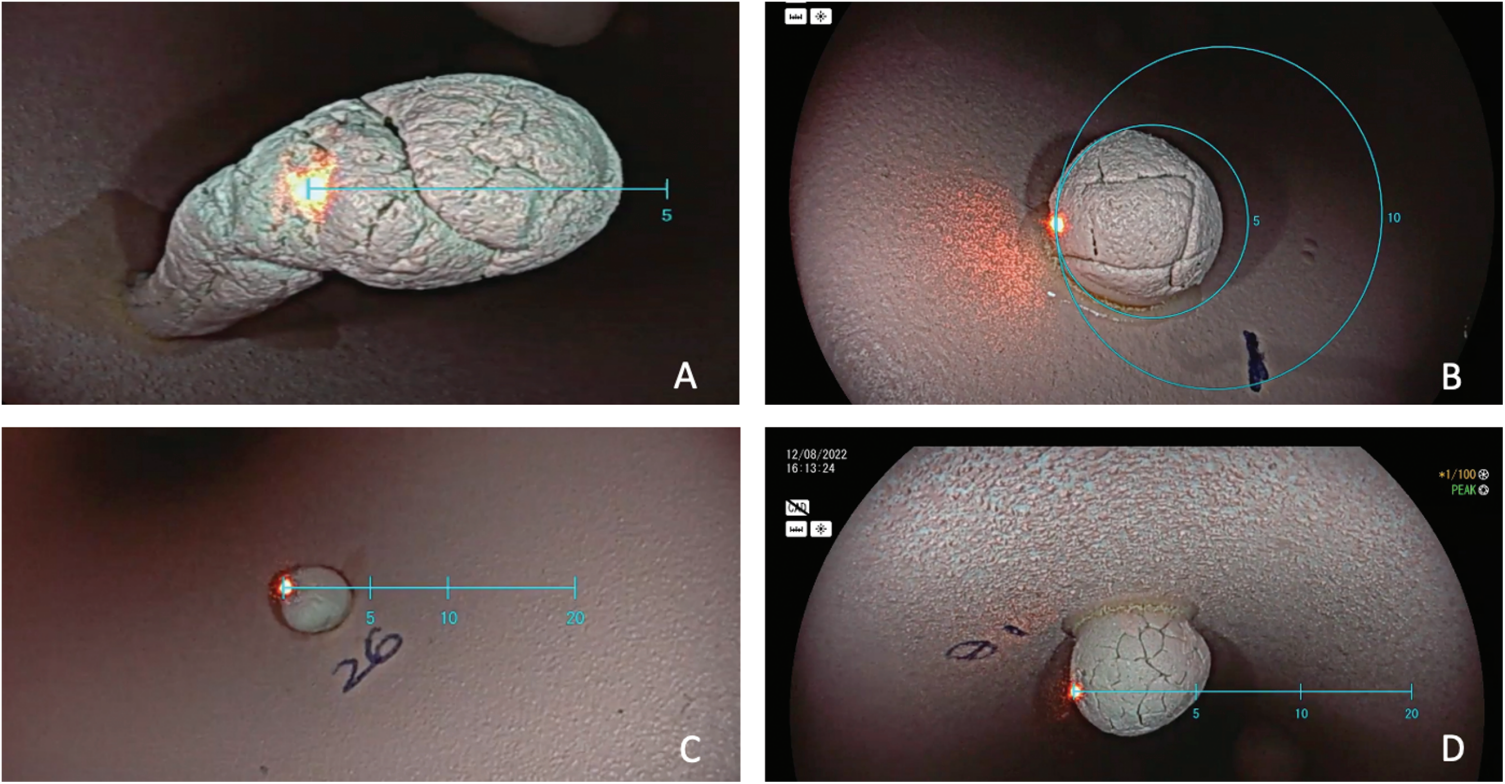
(A) A small pedunculated polyp using the linear scale. (B) A diminutive sessile polyp using the circular scale C. A diminutive sessile polyp using the linear scale B. A small sessile polyp using the linear scale.

A set of 64 artificial polyps was created (fig. 1), designed to be close in size to clinically important size thresholds (3 mm, 5 mm, 10 mm, and 20 mm). These polyps were used for size measurement in a simulated colon model.^3,4^ Polyps were designed to represent test size estimation of clinically relevant size thresholds with varying Paris class morphologies and size groups (diminutive polyps (≤5 mm), small polyps (5–9 mm), polyps at 10–19 mm and polyps (≥20)). Four rounds of size measurements were conducted with different polyp sets. Session 1 (testing 3 mm threshold) had polyps ranging from 1–5 mm, round 2 (testing 5 mm threshold) had polyps ranging from 3 to 7 mm, round 3 (testing 10 mm threshold) had polyps ranging from 8 to 12 mm, and round 4 (testing 20 mm threshold) had polyps ranging from 19 to 22 mm. The selection of these cut-off values was guided by the USMFT and ESGE polypectomy guidelines, ASGE PIVI criteria, as well as various scientific papers that recommend limiting the use of forceps polypectomy to sizes up to 2–3 mm.^5–7^ Each colon contained polyps of three different morphologies: eight sessile (Is), four pedunculated (Ip), and four flat (II-a) (Supplementary material 1). The simulated colon model was a 9 cm rigid tube, simulating a colon in which the four different sets of artificial polyps were placed. The polyps were randomized 1:1 to be measured using either VA or VSE, with the assigned method blinded to the endoscopist until the specific polyp was identified.

### Data set

A total of 383 data points were obtained after these measurements, consisting of 191 VSE measurements and 192 VA measurements. To expand the dataset for size subgroup analysis, we added 480 size measurement datapoints of polyps from two previous publications that used VA or VSE (300 measurements by VSE and 180 measurements by VA).^8,9^ This was done to achieve the required power to compare the relative accuracy (RA) of VSE with VA for all size subgroups.^8,9^ Consequently, the combined dataset comprised a total of 863 measurement datapoints. Methodological consistency was maintained across all three studies by utilizing polyp phantoms made from the same material and using the same simulated colon model and same group of endoscopists.

### Data collection and outcome measures

The study compared VSE and VA’s ability to classify polyps based on size using clinically relevant size thresholds. Polyp misclassification using the true size and the size estimated by these two methods were evaluated. We also evaluated over, and underestimation of polyp measurements defined as polyps that were miscategorized either over or under the true polyp category. Secondary outcomes included RA, stratification based on size and morphology, and time to measure polyps.

### Statistical analysis

SPSS was used for statistical analysis. The study analysed polyps located within 20 percent of each clinically relevant size threshold, namely, ±0.4 mm at 3mm, ±1 mm at 5mm, ±2 mm at 10 mm, and ±4 mm at 20 mm, to compare the ability of VSE and VA in accurately classifying polyps near each threshold. Frequency and proportion were used to define size misclassification. The formula for RA was 100 × (1 - ABSOLUTE VALUE (estimated size - true size)/true size), with means and confidence intervals (CIs) used to represent the data. Mean difference and standard deviation were used for time and size estimation. The proportion of misclassification between VSE and VA was compared using a Chi-squared test with *N*-1 degrees of freedom, and the relative accuracies were compared using an independent *T* test. A *P* value of less than or equal to 0.05 was considered significant.

### Sample size

Previous studies evaluating VSE for size measurement of diminutive polyps have found that the difference in relative size measurement accuracy was 5 percent (79.4 percent for VSE and 74.1 percent for VA).^10,11^ Assuming VSE will have a 79 percent and VA a 74 percent relative size measurement accuracy for diminutive polyps at least 211 diminutive polyp size measurements were required to show a difference of 5 percent in relative size measurement accuracy (80 percent power and an alpha of 0.05).

## Results

### Misclassification of polyps (size)

Overall, VSE had a significantly lower misclassification rate for polyps compared to VA (26 percent versus 48 percent, *P* < 0.0001). When analysing the misclassification rate within clinically relevant size thresholds, VSE had a significantly lower misclassification rate than VA in the 5 mm,10 mm groups (28 percent versus 45 percent, *P* = 0.0159 and 26 percent versus 44 percent, *P* = 0.0135, respectively). However, in the 3 mm and 20 mm groups, VSE had a lower misclassification rate than VA, but the difference was not statistically significant (36 percent vs. 46 percent, *P* = 0.3853 and 38 percent vs. 41 percent, *P* = 0.2705, respectively) (Table 1).

**Table 1.** VSE and visual estimation classification (underestimation, correct, overestimation) based on size category per clinically relevant size threshold.

Threshold (mm)	VSE				VA				Total misclassified VSE versus VA (*P* value)	Underestimation VSE versus VA (*P* value)	Overestimation VSE versus VA (*P* value)
	UE	CC	OE	Total misclassified	UE	CC	OE	Total misclassified			
3 (*n* = 47 VSE) (*n* = 30 visual)	7 (15 percent)	30 (64 percent)	10 (21 percent)	17 (36 percent)	7 (23 percent)	16 (54 percent)	7 (23 percent)	14 (46 percent)	0.3853	0.3772	0.8368
5 (*n* = 105 VSE) (*n* = 83 visual)	14 (13 percent)	74 (72 percent)	17 (15 percent)	31 (28 percent)	20 (24 percent)	46 (55 percent)	17 (21 percent)	37 (45 percent)	0.0159	0.0511	0.2852
10 (*n* = 102 VSE) (*n* = 72 visual)	19 (19 percent)	75 (74 percent)	8 (7 percent)	27 (26 percent)	27 (38 percent)	40 (56 percent)	5 (6 percent)	32 (44 percent)	0.0135	0.0055	0.7940
20 (*n* = 97 VSE) (*n* = 89 visual)	21 (22 percent)	60 (62 percent)	16 (16 percent)	37 (38 percent)	40 (45 percent)	48 (54 percent)	1 (1 percent)	41 (46 percent)	0.2705	0.0009	0.0003
Total (*n* = 491 VSE) (*N* = 372 Visual)	69 (14 percent)	363 (74 percent)	59 (12 percent)	128 (26 percent)	118 (32 percent)	213 (57 percent)	41 (11 percent)	159 (43 percent)	<0.0001	<0.0001	0.6494

UE, underestimation; OE, overestimation; CC, correct classification, VSE, virtual scale estimation; VA, visual assessment.

### Over and under-estimation of polyp size

Overall, VA underestimated polyp size more frequently than VSE, with rates of 32 percent and 14 percent, respectively, at *P* < 0.0001. When assessing clinically relevant size thresholds (3 mm, 5 mm, 10 mm, and 20 mm), VA consistently underestimated more often than VSE at all thresholds (23 percent vs. 15 percent at 3 mm at *P* = 0.3772; 24 percent vs. 13 percent at 5 mm at *P* = 0.05; 38 percent vs. 19 percent at 10 mm at *P* = 0.0055; and 45 percent vs 22 percent at 20 mm at *P* = 0.0009) as shown in Table 1. The results of the study indicate that the VSE method had an overestimation frequency of 21 percent when using a size threshold of 3 mm, 15 percent at a threshold of 5 mm, 7 percent at a threshold of 10 mm, and 16 percent at a threshold of 20 mm. Conversely, the VA method had an overestimation frequency of 27 percent at a 3 mm threshold, 18 percent at a 5 mm threshold, 6 percent at a 10 mm threshold, and only 1 percent at a 20 mm threshold (Table 1).

### Performance for polyp measurements

The study found that when comparing VSE to VA for measuring polyp size, VSE had a higher RA of 80.6 percent (95 percent CI: 80.3–83.0) compared to 69.4 percent (95 percent CI: 66.1–72.4) for VA. Additionally, VSE was found to be significantly more accurate than VA when measuring polyps in all size ranges. Specifically, between 0 and 4.99 mm (76.3 percent (95 percent CI: 72.5–79.6) to 63.3 percent (95 percent CI: 53.5–70.7)) with *P* = 0.0039, 5–9.99 mm (82.3 percent (95 percent CI: 79.9–84.5) to 65.9 percent (95 percent CI: 64.4–72.3)) with *P* < 0.0001, 10–19.99 mm (83.8 percent (95 percent CI: 81.7–85.8) to 65.9 percent (95 percent CI: 58.8–71.8) and >20 mm (85.1 percent (95 percent CI: 82.4–87.5) to 73.3percent (95 percent CI: 69.0–77.3)) with *P*< 0.0001 (Table 2).

**Table 2.** Relative accuracy of measurement by each tool against true measurement per polyp size interval (comparing VSE to visual).

Relative accuracy of measurement by each tool against true measurement (percent) (95 percent CI)	VSE	VA	*P* value
All polyps	80.6 (80.3–83.0)	69.4 (66.1–72.4)	<0.0001
*Polyp size*			
0–4.99 mm	76.3 (72.5–79.6)	63.3 (53.5–70.7)	0.0039
5–9.9 mm	82.3 (79.9–84.5)	65.9 (58.8–71.8	<0.0001
10–19.9 mm	83.8 (81.7–85.8)	65.9 (64.4–72.3)	<0.0001
≥20 mm	85.1 (82.4–87.5)	73.3 (69.0–77.3)	<0.0001

CI, confidence interval; VSE, virtual scale estimation; VA, visual assessment.

### Time for size estimation using each method

On average, endoscopists took approximately 25 s longer to measure polyps using VSE, as compared to VA (*P* < 0.0001) (Table 3).

**Table 3.** Time of polyp measurement VSE versus VA.

	Average time in seconds (std. dev)		
Polyp measurement modality	VSE	VA	Comparing VSE and VA
	37.3 S (27.0)	12.2 S (10.1)	*P* < 0.0001

VSE, virtual scale estimation; VA, visual assessment.

## Discussion

We found in this study that VA significantly more often misclassifies polyps at clinically relevant size thresholds 5 mm and 10 mm compared to VSE. These size thresholds are vital during colonoscopy since follow-up recommendations can differ significantly based on adenomatous polyp size.^5,10^ For example, guidelines recommend follow-up colonoscopy changes from 10 years to 3 years if a tubular adenoma is judged to be less than or greater than 10 mm.^5^ The 5 mm threshold is important for implementation of the resect and discard strategy. Since using VSE improves the classification of diminutive polyps it may give endoscopists more confidence in implementing this strategy. Our study suggests that VSE might significantly improve clinical decision-making by correctly assigning polyp into size groups above or below 5 and 10 mm.

Our analysis of the underestimation of polyp sizes, based on clinically relevant thresholds, revealed that VA had a significantly higher frequency of underestimation overall than VSE (32 percent and 14 percent, respectively, at *P* < 0.0001). We showed a statistically significant difference in underestimation at the 5 mm, 10 mm, and 20 mm clinically relevant size thresholds, but this difference was not significant at the 3 mm threshold. VSE is more accurate and avoids underestimation seen with VA at important size thresholds, reducing the risk of non-identification of precancerous lesions and improving surveillance accuracy.

Our study demonstrated that VSE had significantly greater RA across all size subgroups (≤5 mm, 5–9 mm, 10–19 mm, and ≥20 mm) compared to VA. Notably, we are the first to report a statistically significant difference in the RA of diminutive polyps (<5 mm), which had not been shown in previous studies. Our results add to the existing literature and provide further evidence of the advantages of VSE over VA in accurately identifying polyp sizes, particularly for diminutive polyps. Two studies found that mis-sizing polyps may lead to up to 11 percent incorrect surveillance intervals.^2,12^ Utilizing VSE for improved accuracy has the potential to enhance correct surveillance interval assignment.

This study has several strengths that contribute to the validity of its findings. First and foremost, the large sample size of 863 measured polyps. Furthermore, while previous publications have compared the misclassification of polyps using VSE and VA,^8,9^ our study is distinct in that we analyse polyps near clinically relevant size thresholds. Some limitations need to be mentioned. The increased time required to measure the polyps with VSE may have contributed to an increase in measurement accuracy, as the additional time allowed for more precise and careful measurements. Incorporating data from previous studies allowed us to increase the power of our study, but it also introduced the potential for selection bias. To mitigate this risk, we used data from two studies with similar protocols that involved the same endoscopists and materials for measuring polyps with VA and VSE. This approach helped to minimize the potential for bias. To further evaluate the reproducibility of our study, it would have been valuable to include a comparison of VSE to VSE, performed as a randomized crossover study in addition to the comparison of VSE to VA. However, due to the constraints of resources and time in our trials, we were unable to include this method. Our study revealed no statistically significant difference in the misclassification of VSE compared to VA for polyps at the 20 mm cut off. However, it is important to acknowledge that our study had a limitation in that the maximum diameter for polyps exceeding 20 mm was only 22 mm. Although this limitation exists, we hypothesize that the lack of statistical power at this clinically significant size threshold is a more plausible explanation. Thus, in future studies, we will address this limitation by including larger 3D-printed polyps. An important endpoint is assessing how accurate polyp size measurement and misclassification affect colonoscopy surveillance intervals. While it is impossible to calculate intervals in this pre-clinical trial without pathology data, initial clinical studies using VSE suggest that better size measurement accuracy impacts interval assignment based on guidelines.^13^

In this randomized, blinded pre-clinical trial, our findings demonstrate that VSE outperforms VA in categorizing polyps by size, particularly at the clinically relevant size thresholds of 5 mm and 10 mm. Our study also revealed that VSE has a significantly higher RA for diminutive polyps compared to VA, and this RA is maintained across all size categories.

## Supplementary material

Supplementary material is available at Journal of the Canadian Association of Gastroenterology online.

gwad057_suppl_Supplementary_Material

## Data Availability

The data underlying this article will be shared on reasonable request to the corresponding author.
