# Supplementary material for: A Preclinical Blinded Randomized-Controlled Trial Evaluating the Clinical Relevance of Polyp Size Measurement Using a Virtual Scale Endoscope
Source: J Can Assoc Gastroenterol. 2023 Dec 23;7(2):149–53. doi: 10.1093/jcag/gwad057 (PMC10999760; doi:10.1093/jcag/gwad057)
Supplement: gwad057_suppl_Supplementary_Material [file gwad057_suppl_supplementary_material.zip › online-only supplement (scale eye).docx]

*Participants*

Six endoscopists from the CHUM estimated the size of all simulated polyps. Three of the endoscopists were experienced gastroenterologists (DvR, BP, SS) with over 5000 completed colonoscopies, and the remaining three were gastroenterology residents (RD, MAK, JL) with 100-500 completed colonoscopies.

*Polyp characteristics*

Within the combined data set, polyps were characterized into four groups based on size and five groups based on Paris class morphology. 233 polyps between 0 - 4.9 mm were measured (106 by VA and 127 by VSE), 256 polyps between 5 - 9.99 mm (105 by VA and 151 by VSE), 224 polyps between 10 - 19.99 mm (93 by VA and 131 by VSE) and 150 polyps greater than 20 mm (largest being 22 mm) (68 by VA and 82 by VSE). 401 polyps classified as Paris Is were measured (157 by VA and 244 by VSE), 222 polyps as Paris Ip (114 by VA and 108 by VSE), 230 polyps as Paris II-A (101 by VA and 129 by VSE), 9 polyps as Paris II-c (0 by VA and 9 by VSE) and 1 polyp as Paris Isp (0 by VA and 1 by VSE). **(Table 1.1)**

Table 1.1. # polyps per size category and Paris classification

|  | **VA** | **VSE** | **Total** |
| --- | --- | --- | --- |
|  | **(# of polyps)** | **(# of polyps)** | **(# of polyps)** |
| **All polyps** | 372 | 491 | 863 |
| **Polyp size** |  |  |  |
| **0-4.99 mm** | 106 | 127 | 233 |
| **5-9.9mm** | 105 | 151 | 256 |
| **10-19.9mm** | 93 | 131 | 224 |
| **≥20mm** | 68 | 82 | 150 |
| **Paris Class** |  |  |  |
| **Is** | 157 | 244 | 401 |
| **Ip** | 114 | 108 | 222 |
| **II-a** | 101 | 129 | 230 |
| **II-c** | 0 | 9 | 9 |
| **Isp** | 0 | 1 | 1 |

VSE: Virtual Scale Estimation

VA: Visual Assessment
